# Volatiles Emitted from Maize Ears Simultaneously Infected with Two *Fusarium* Species Mirror the Most Competitive Fungal Pathogen

**DOI:** 10.3389/fpls.2016.01460

**Published:** 2016-09-27

**Authors:** Mohammed Sherif, Eva-Maria Becker, Cornelia Herrfurth, Ivo Feussner, Petr Karlovsky, Richard Splivallo

**Affiliations:** ^1^Molecular Phytopathology and Mycotoxin Research, University of GöttingenGöttingen, Germany; ^2^Integrative Fungal Research ClusterFrankfurt, Germany; ^3^Institute for Molecular Biosciences, University of FrankfurtFrankfurt am Main, Germany; ^4^Department of Plant Biochemistry, Albrecht-von-Haller-Institute for Plant Sciences, University of GöttingenGöttingen, Germany

**Keywords:** maize, Zea mays, volatile organic compounds, sesquiterpenoids, *Fusarium* spp., fungal pathogens, oxylipins

## Abstract

Along with barley and rice, maize provides staple food for more than half of the world population. Maize ears are regularly infected with fungal pathogens of the *Fusarium* genus, which, besides reducing yield, also taint grains with toxic metabolites. In an earlier work, we have shown that maize ears infection with single *Fusarium* strains was detectable through volatile sensing. In nature, infection most commonly occurs with more than a single fungal strain; hence we tested how the interactions of two strains would modulate volatile emission from infected ears. For this purpose, ears of a hybrid and a dwarf maize variety were simultaneously infected with different strains of *Fusarium graminearum* and *F. verticillioides* and, the resulting volatile profiles were compared to the ones of ears infected with single strains. Disease severity, fungal biomass, and the concentration of the oxylipin 9-hydroxy octadecadienoic acid, a signaling molecule involved in plant defense, were monitored and correlated to volatile profiles. Our results demonstrate that in simultaneous infections of hybrid and dwarf maize, the most competitive fungal strains had the largest influence on the volatile profile of infected ears. In both concurrent and single inoculations, volatile profiles reflected disease severity. Additionally, the data further indicate that dwarf maize and hybrid maize might emit common (i.e., sesquiterpenoids) and specific markers upon fungal infection. Overall this suggests that volatile profiles might be a good proxy for disease severity regardless of the fungal competition taking place in maize ears. With the appropriate sensitivity and reliability, volatile sensing thus appears as a promising tool for detecting fungal infection of maize ears under field conditions.

## Introduction

Maize fields cover about 180 million hectares worldwide and provide, along with wheat and rice, staple food for more than half of the world population (FAO, 1995). Maize cultivation suffers from numerous pathogens, which infect plant roots, stems, leaves, and ears in the field. Some of the most devastating pathogens of maize belong to the *Fusarium* genus which is responsible for 10–30% yield loss in major crops throughout the globe (Agrios, 2005). Maize ear infection is typically caused by a mixture of *Fusarium* species (Kedera et al., 1994; Doohan et al., 2003), the most common of which are *Fusarium graminearum* Schwabe and *F. verticillioides* (Sacc.) Nirenberg (Vigier et al., 2001; Logrieco et al., 2002). Apart from causing yield losses, *Fusarium* species infecting maize produce mycotoxins potentially endangering the health of consumers and farm animals.

Controlling and detecting early infection of maize by *Fusarium* spp. is challenging. Disease symptoms may become visible at late stages of infection because the pathogen infect kernels through the rachis (Oldenburg and Ellner, 2015), or the infection may even proceed without visible symptoms (Bacon and Hinton, 1996). Serological and molecular diagnostic techniques require sample destruction and are therefore not suitable for real-time monitoring (Nezhad, 2014). Volatile sensing has emerged as a promising alternative to detect disease in crops (Sankaran et al., 2010; Aksenov et al., 2013). The rationale for volatile sensing is that the volatile blend emitted by plants depends on their physiological status, which is affected by the presence of a pathogen. Comparison of volatile profiles of infected and non-infected plants might allow the identification of volatile biomarkers that can be used to monitor fungal infection in real time using non-invasive techniques.

Most volatiles emitted by plants and microbes are secondary metabolites with low molecular weight of a lipophilic nature and a high vapor pressure (Dudareva et al., 2006; Lemfack et al., 2014). To date nearly 2,000 volatiles have been described in plants (Knudsen et al., 2006; Dunkel et al., 2009; Schenkel et al., 2015), while a little more than 1,000 volatile compounds have been documented from bacteria and fungi (Lemfack et al., 2014; Schenkel et al., 2015). Most of these volatiles are terpenoids, phenylpropanoids/benzenoids, fatty acid, and amino acid derivatives (Dudareva et al., 2004). Volatile metabolites mediate ecological interactions among plants, microbes and other organisms and may thus affect defense against pathogens and herbivores (Piesik et al., 2013; Peñuelas et al., 2014; Kanchiswamy et al., 2015; Schenkel et al., 2015). Technically, volatiles can also be considered as indicators for the physiological status of the plant (Baldwin, 2010; Wenke et al., 2010; Clavijo McCormick et al., 2012). For example Jansen et al. (2009) showed that tomato plants infected with the fungal pathogen *Botrytis cinerea* released higher quantities of mono- and sesquiterpenes than their healthy counterparts. The alcohols 1-penten-3-ol and (3*Z*)-hexen-1-ol are induced in chickpea infected with the fungal pathogen *Ascochyta rabiei* (Cruz et al., 2012). We have similarly demonstrated that the emission of 22 volatiles was regulated in maize ears infected with single strains of *Fusarium* spp. fungal pathogens (Becker et al., 2013, 2014). The most common biomarkers of *Fusarium* spp. infection were the sesquiterpenoids β-macrocarpene and β-bisabolene, however, some other markers (octan-3-ol and β-farnesene) were strain specific (Becker et al., 2013, 2014).

Here, we extend our investigation to the volatile profiles of maize ears simultaneously and separately infected with *F. graminearum* and *F. verticillioides*, using strains that differ in their aggressiveness toward maize. We wanted to know if simultaneous infection would lead to a volatile profile which differed from single infections. For this purpose we concurrently inoculated maize ears with strains of *F. graminearum* and *F. verticillioides* and compared their volatile profiles to the one of ears infested with single fungal strains. We also monitored fungal biomass and disease severity and overall interpret shifts in volatile profiles in light of competitive fungal interactions.

## Materials and Methods

### Fungal Species

Seven strains belong to *F. graminearum* and *F. verticillioides* were used (**Table 1**). Sporulation was achieved on Mung bean medium (Bai and Shaner, 1996; Becker et al., 2014). Spore density was determined using a Thoma chamber (0.0025 mm^2^) and adjusted to the desired concentrations in sterile water. Spore viability was checked on potato dextrose agar (PDA).

**Table 1:** Fungal strains of *Fusarium graminearum* and *F. verticillioides* used in maize ear infections.

Fungal strain	Name	Abbreviation	Source
*F. graminearum*	Fg71^a^	FG1	T. Miedaner, University of Hohenheim, Germany
*F. graminearum*	Fg210.1 wt^b^	FG2	Phytopathological strain collection, Division of Plant Pathology and Crop Protection, Georg-August-University Göttingen, Germany
*F. graminearum*	FG 06^a^	FG3	Wilhelm Schäfer, Hamburg University, Hamburg, Germany
*F. graminearum*	FG 2311^b^	FG4	
*F. verticillioides*	Fv Ita 1^c^	FV1	A. Prodi, University of Bologna, Italy
*F. verticillioides*	FM8114^c^	FV2	Fusarium Research Centre, Pennsylvania State University, USA
*F. verticillioides*	M-3125^c^	FV3	Robert Proctor, National Center for Agricultural Utilization Research/U.S. Department of Agriculture Peoria, IL, USA


*^a^Nivalenol-producing fungal strain, (NIV chemotype). ^b^Deoxynivalenol-producing fungal strain, (DON chemotype). ^c^Fumonisin-producing fungal strain.*

### Plant Material and Cultivation

Two maize (*Zea mays* L.) varieties were employed here, the hybrid field variety Ronaldinio (KWS Saat AG, Einbeck, Germany) and the dwarf maize variety Gaspe Flint (collected in Quebec, Canada). Maize kernels were surface sterilized with 4% aqueous solution of sodium hypochlorite for 15 min and rinsed three times with sterile water. Kernels were planted into autoclaved soil (topsoil/sand; 2:1 v/v) filled in plastic pots. Seedlings were grown in a greenhouse (26 ± 4°C, 14 h photoperiod) until full development of the maize ears and fertilized as required using mineral fertilizer Hakaphos^®^ (COMPO Expert GmbH , Münster, Germany).

### Fungal Inoculation of Maize Ears

Hybrid and dwarf maize plants were infected at the main flowering stage either with a single strain or simultaneously with two *Fusarium* strains as a 50:50 mixture (**Table 2**). This time point corresponds to approximately 4 and 7 days after silking for the dwarf maize and hybrid maize, respectively. The concentration of inoculated spores was adjusted to approximately 10^5^ or 10^6^ spores/mL according to spores’ viability and a volume of 0.5 mL (dwarf maize) or 1.0 mL (hybrid maize) inoculum were injected into the silk channel (**Table 2**). Mock inoculation with sterile water was used as a control. All treatments for fungal biomass quantification and volatile profiling were replicated on four plants (hybrid maize) and five plants (dwarf maize). Oxylipins were quantified from hybrid maize using five ears (replicates) from control/uninfected plants, four to seven replicates for single inoculations with strains FG1, FG2, FV1, FV2; and seven replicates for each of the mixed inoculations FG1+FV1 or FG2+FV2.

**Table 2:** Infection of maize plants with *Fusarium* species.

Treatment	Fungal strain	Spore concentration	Volume	Host plant
Single	FG1	10^5^ mL^-1^	1.00 mL	Hybrid maize
	FG2	10^5^ mL^-1^	1.00 mL	Hybrid maize
	FG3	10^5^ mL^-1^	0.50 mL	Dwarf maize
	FG4	10^5^ mL^-1^	0.50 mL	Dwarf maize
	FV1	10^6^ mL^-1^	1.00 mL	Hybrid maize
	FV2	10^6^ mL^-1^	1.00 mL	Hybrid maize
	FV3	10^5^ mL^-1^	0.50 mL	Dwarf maize
Mix	FG1+FV1	10^5^ mL^-1^ (50:50)	1.00 mL	Hybrid maize
	FG2+FV2	10^5^ mL^-1^ (50:50)	1.00 mL	Hybrid maize
	FG3+FV3	10^5^ mL^-1^ (50:50)	0.50 mL	Dwarf maize
	FG4+FV3	10^5^ mL^-1^ (50:50)	0.50 mL	Dwarf maize


### Assessing Disease Severity and Sampling of Ears

Disease symptoms on infected maize ears were indexed 24 and 18 days post fungal inoculation in hybrid and dwarf maize varieties, respectively. The dehusked maize ears showing infection symptoms (i.e., fungal mycelium and/or rotting) were graded on an index scale from zero to eight as described earlier (Sherif et al., unpublished). Ear kernels were cut off and immediately collected for volatile profiling, fungal DNA quantification and oxylipin analysis as described in Becker et al. (2014).

### Fungal Biomass Quantification

DNA was extracted from aliquots of 100 mg maize flour following a protocol of Brandfass and Karlovsky (2008). Fungal DNA was quantified in the samples (10–15 ng μL^-1^) by qPCR using species specific primers for *F. graminearum* (Nicholson et al., 1998) and *F. verticillioides* (Mulè et al., 2004). DNA from control (uninfected) ears were also subjected to qPCR using the aforementioned primers to ascertain that there were not contaminated control plants.

### Oxylipin Analysis

Aliquots of freeze-dried maize material, corresponding to 2.0 g fresh weight, were extracted according to the protocol of Gobel et al. (2003) and methylated with trimethylsilyl diazomethane (2 M in hexane, Sigma-Aldrich, Taufkirchen, Germany). As an internal standard, (6*Z*,9*Z*,11*E*,13*S*)-13-hydroxy-6,9,11-octadecatrienoic acid was added. Hydroxyl fatty acids were purified on reverse phase-HPLC equipped with ET250/2 Nucleosil 120-5 C18 column (Macherey-Nagel, Dueren, Germany) as described in Gobel et al. (2003). Eluate fraction was collected between 8 and 13.5 min, evaporated to dryness and re-dissolved in 2 μL acetonitrile. After addition of 2 μL N,O-bis(trimethylsilyl)trifluoroacetamide (Sigma-Aldrich, Taufkirchen, Germany), analysis was carried out with an Agilent 6890 gas chromatograph equipped with a capillary DB-23 column (Agilent, Waldbronn, Germany, nominal diameter: 0.25 mm, length: 30 m, nominal film thickness: 0.25 μm) and coupled with an Agilent 5973 MS. Standard curves were constructed by plotting ion intensities vs. molar amounts of known hydroxyl fatty acids.

### Full Volatile Profiling

The samples of 2.0 g kernels were enclosed in 20 mL solid-phase microextraction (SPME) vials sealed air tight with a screw cap containing a silicon/polytetrafluoroethylene septum. Samples were extracted for 10 min at 40°C using a 1.0 cm SPME (PDMS/DVB fiber) and, for the hybrid maize samples, volatile were profiled as described in Becker et al. (2014). For the dwarf maize variety, the temperature programming of the GC oven was modified to achieve a better separation of volatiles compared to Becker et al. (2014). Specifically the following parameters were used: 40°C for 3 min, increasing at 1.5°C min^-1^ to 80°C, followed by 80°C min^-1^ to 250°C (7.21 min isothermic).

GC/MS output data was processed using two different approaches. TagFinder version 4.1 (Luedemann et al., 2008) was used for the dwarf maize data set with the following parameters; Timescale: 2, Low Mass: 40, High Mass: 400. Peakfinder tool; SmoothWidth Apex Finder: 1, Low Intensity Threshold: 20000 (non-Smooth Apex), Smooth Width +/- Apex Scan: 1 (non-Merge Peaks). Peak alignment; Time ScanWidth 4.0; Gliding Median Group Count 1; Min Fragment Intensity 50. Volatile profiles of hybrid maize were processed as described in Becker et al. (2014).

Volatiles were identified using Kovats retention indices, the NIST 2008 Mass Spectral library (version 2.0f), the ADAMS MS library (Adams, 2005), and authentic standards when available, specifically for: pentane, dimethyl sulfide, 3-methyl-butanal, 2-methyle-butanal, 3-hexene-1-ol, heptan-2-ol, octan-3-ol, 1-octen-3-ol, octan-3-one, α-selinene, β-selinene, β-bisabolene, β-macrocarpene.

### Statistical Analysis

Statistical analysis was performed using the statistics software PAST version 3.04 (Hammer et al., 2001) for the principal component analysis (PCA) and cluster trees. Disease severity and fungal DNA (log transformed values) were compared among treatments with Tukey’s pairwise test (PAST version 3.04). In addition, the non-parametric Kruskal-Wallis test performed in R, version 3.0.3 (R Development Core Team, 2008) was used for both maize varieties to identify volatile markers that significantly differed among treatments (i.e., control plants, plants infected with one fungus, plants infected with two fungi).

## Results

Two maize varieties, including a hybrid variety with wide commercial usage and a dwarf maize variety with short life cycle were selected for our experiments. Hybrid and dwarf maize were (i) infected with single strains of *F. graminearum* and *F. verticillioides*, (ii) simultaneously infected with different strains belonging to the two aforementioned species, and (iii) uninfected (“mock-inoculated” with water). All strains used in this work are listed in **Table 1**, and specific combinations of maize varieties and strains are listed in **Table 2.** In short, hybrid maize was infected with either *F. graminearum* (FG) strain FG1, *F. verticillioides* (FV) strain FV1 and mixed strains FG1+FV1 or with FG2, FV2, and mixed strains FG2+FV2. By contrast dwarf maize was infected with strains FG3, FV3 and mixed strains FG3+FV3 or with strain FG4 and mixed strains FG4+FV3. Volatiles were profiled in all cases by SPME-GC/MS, submitted to statistics to identify infection biomarkers and highlight trends in the data.

### Volatile Profiles in Mixed Inoculations Are Governed by the Most Competitive Fungal Strain

**FIGURE 1:**
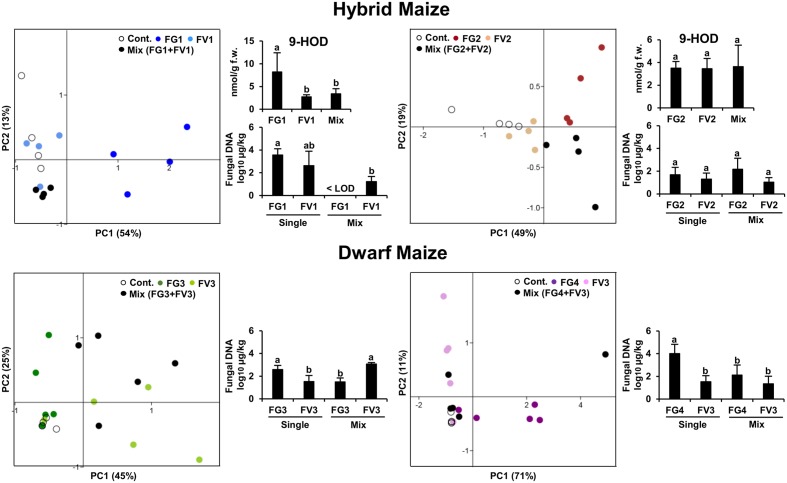
**Principle Component Analysis (PCA) of the volatile infection biomarkers in infected and uninfected maize ears for hybrid and dwarf maize.** Each dot represents one replicate from each treatment. 9-HOD: (10*E*,12*Z*)-9-hydroxy-10,12-octadecadienoic acid. FW, fresh weight. Different letters indicate statistical differences (*p* < 0.05) Tukey’s pairwise test.

Our first aim was to understand how competitive interactions between two *Fusarium* species affected the volatile profiles of hybrid and dwarf maize. For this purpose, PCA was performed on the volatile biomarkers of hybrid and dwarf maize, considering always four groups of samples made of (1) uninfected ears, (2) infected with *F. graminearum*, (3) infected with *F. verticillioides*, and (4) infected with both species. Two different strain combinations were used for hybrid maize and two others for dwarf maize, resulting in four PCAs as shown in **Figure 1.** Depending on the cases, PCA could explain from 67–82% data variability in terms of volatile profiles (equivalent to the sum of the scatter plot scores for both axis/principal components PC1 and PC2).

In the case of hybrid maize, volatile profiles of single inoculations differed the most from uninfected ears for strains FG1 and FG2 (**Figure 1**), despite the fact that the latter strains accumulated comparable biomass to strains FV1 and FV2, respectively. In simultaneous infections, the volatile profile of mixed inoculation FG1+FV1 was somehow comparable to the one of the single inoculation FV1, possibly reflecting the drop in biomass of FG1 combined with FV1 compared to single inoculation FG1. A comparable trend was observed for the concentration of the oxylipin 9-hydroxy octadecadienoic acid (9-HOD). Indeed its concentration in the simultaneous inoculation with FG1+FV1 was similar to the one of the single inoculation with FV1. The volatile profile of mixed inoculation FG2+FV2 differed from the one of single inoculations with the same strains, while fungal biomass and the concentration of 9-HOD remained unaffected (**Figure 1**).

In the case of dwarf maize, volatile profiles of single inoculations differed the most from uninfected ears for strains FV3 and FG4 (**Figure 1**) and, the highest biomass was reached by FG3 and FG4. In simultaneous infections, the volatile profile of mixed inoculation FG3+FV3 was half-way between the one of maize ears inoculated with single strains, and biomass accumulation was stimulated for FV3 and inhibited for FG3 compared to single inoculation with the same strain. The profile of mixed inoculation FG4+FV3 partially overlapped with the one of maize ears inoculated with single strains, which however, displayed important data variability. In terms of biomass, FV3 was unaffected, however, FG4 was inhibited compared to single inoculations.

Overall the data indicates that volatile profiles in mixed inoculations are governed by the most competitive fungal strain, and this does not correlate with their ability to produce a specific mycotoxin.

### In Both Concurrent and Single Inoculations, Volatile Profiles Reflect Disease Severity

**FIGURE 2:**
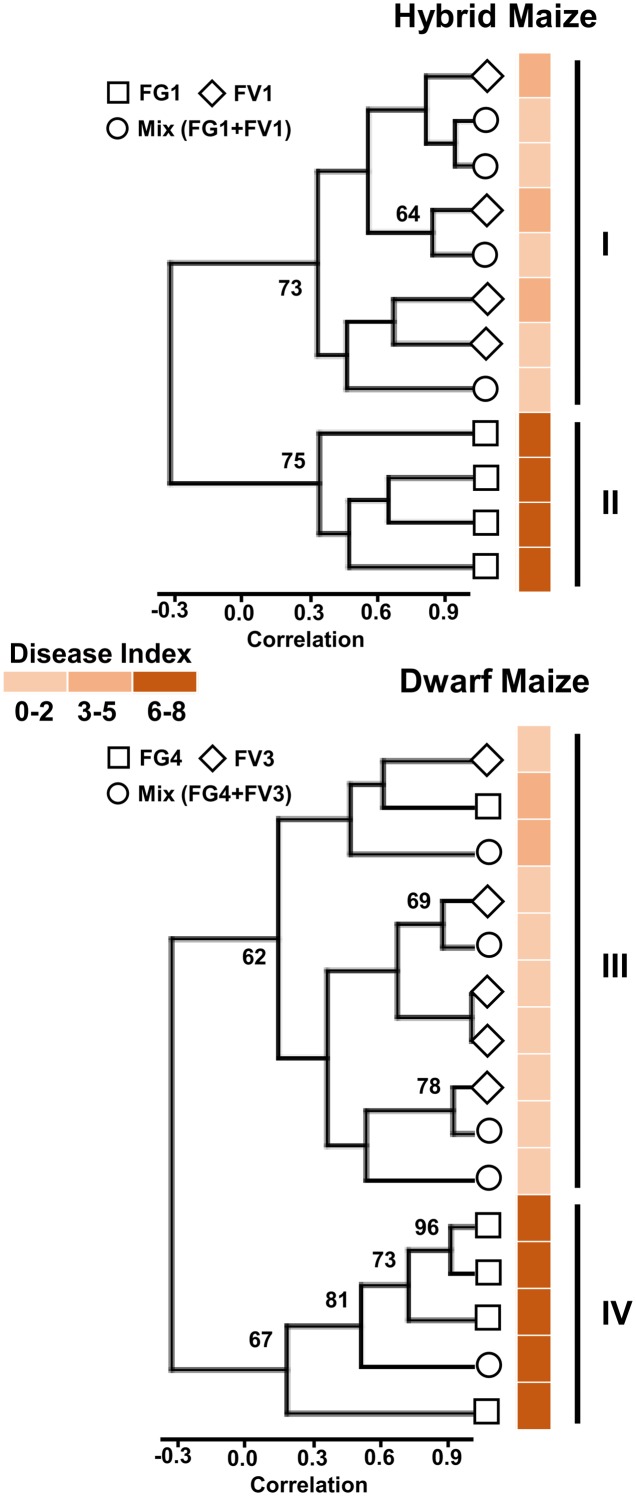
**Cluster tree based on volatile infection biomarkers of maize ears infected with one or two *Fusarium* species.** Bootstrap values >60% are indicated (*N* = 10,000 bootstraps) on the tree along with disease index (color coded) for each individual samples (maize ears).

In order to investigate a possible correlation between volatile profiles and disease severity, we applied cluster analysis to the volatile biomarkers of hybrid and dwarf maize and displayed the resulting analysis along with disease severity for each treatment and replicate. Results are shown in (**Figure 2**) for hybrid maize and dwarf maize. Considering clusters with boot-strap values larger than 60%, two major clusters are visible for both maize varieties. For hybrid maize, one cluster includes all samples infected with FV1, either alone or with FG1 (FG1+FV1; **Figure 2**, cluster I), while the other cluster includes all replicates infected with FG1 (**Figure 2**, cluster II). For dwarf maize, one cluster includes all replicates infected with FV3 alone, four of five replicates simultaneously infected with FG4+FV3 and one replicate infected with FG4 (**Figure 2**, cluster III), the other cluster includes four of five replicates infected with FG4 and one replicate simultaneously infected with FG4+FV3 (**Figure 2**, cluster IV). Disease index depicted on the right side of the diagrams highlight that the clustering is dependent on disease severity for both hybrid and dwarf maize (**Figure 2**).

We further investigated the correlation between disease severity and the oxylipin 9-HOD (**Figure 1**). Disease severity and the concentrations of 9-HOD quantified in hybrid maize were significantly correlated [*p* < 0.05, two-tailed *t-*test computed in PAST version 3.04 (Hammer et al., 2001)]. Considering the overall data, *R*^2^ = 0.49 (*p* < 0.05). Considering inoculations with single strains and uninfected controls, *R*^2^ = 0.67 (*p* < 0.05). Considering mixed inoculations and uninfected controls, *R*^2^ = 0.23 (*p* < 0.05).

In summary our results exemplify that the overall volatile profile of maize ears reflects disease severity regardless of the presence of one or more *Fusarium* species, and highlight that the correlation between disease severity and oxylipin concentrations (9-HOD) is higher in single inoculations compared to mixed inoculations.

### Dwarf Maize and Hybrid Maize Share Common and Specific Volatile Infection Markers

**FIGURE 3:**
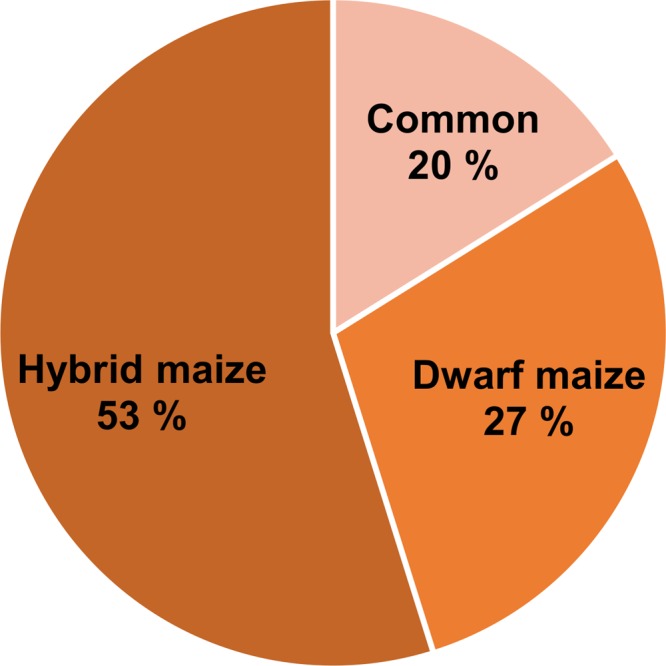
**Pie chart representing the percentage of common and specific infection biomarkers to hybrid and dwarf maize.** The numbers take into account identified, tentatively identified, and unidentified volatiles.

**FIGURE 4:**
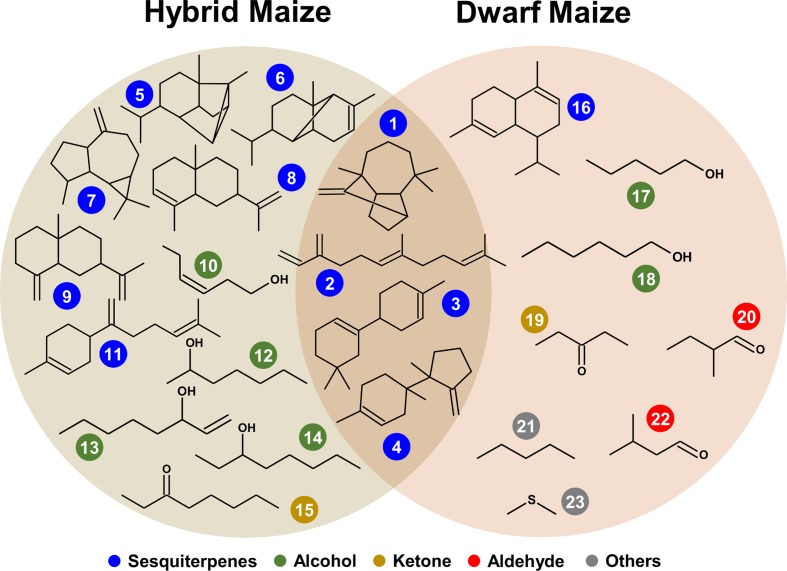
**Structures of common and specific infection biomarkers in hybrid and dwarf maize.** Color coder represent chemical classes: (1) (+)-longifolene, (2) β-farnesene, (3) β-macrocarpene, (4) trichodiene, (5) (+)-cycloisosativene, (6) α-ylangene, (7) (+)-aromadendrene, (8) α-selinene, (9) β-selinene, (10) 3-hexen-1-ol, (11) β-bisabolene, (12) heptan-2-ol, (13) 1-octen-3-ol, (14) octan-3-ol, (15) octan-3-one, (16) α-muurolene, (17) pentan-1-ol, (18) hexan-1-ol, (19) pentan-3-one, (20) 2-methyl-butanal, (21) pentane, (22) 3-methyl-butanal, (23) dimethyl sulfide.

Volatile compound identification was achieved using Kovats retention indices, mass spectral libraries, and authentic standards when available. VOC markers which concentration significantly differed between healthy and infected plants included an alkane, a sulfur compound, alcohols, ketones, and terpenoids and some unidentified compounds. From both maize varieties, 23 volatile markers could be identified or tentatively identified, 12 from dwarf and 15 from hybrid maize, and both varieties shared six common markers including; (+)-longifolene, β-farnesene, β-macrocarpene, trichodiene, and two unidentified SQT (**Figures 3** and **4**). The pie chart in **Figure 3** illustrating the number of volatiles common and specific to both maize varieties includes unidentified volatiles in addition to the identified and tentatively identified ones listed in **Figure 4.** Differences in the volatile markers of both maize varieties could be ascribed to aldehydes, one alkane and a sulfur compound present in dwarf maize only whereas numerous sesquiterpenoids could solely be detected from hybrid maize (**Figure 4**).

**FIGURE 5:**
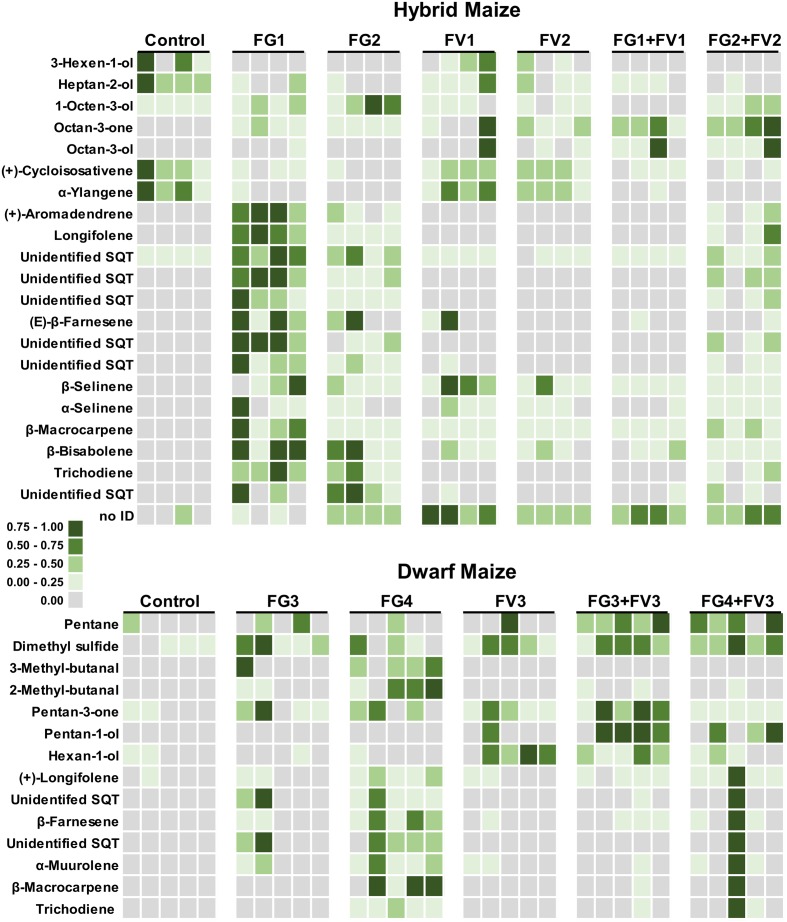
**Heatmap representing volatiles that are regulated in dwarf and hybrid maize upon infection with *Fusarium*.** Squares correspond to the concentration of single volatiles emitted from independent ears for each treatment – dwarf maize, *n* = 5 replicates per treatment; hybrid maize, *n* = 4 replicates per treatment. Squares have been color coded to represent volatile concentrations (normalized from zero to one). The heatmap illustrates that volatiles are differentially regulated by single inoculations or co-inoculations of *Fusarium* strains. Treatments: Control, uninfected ears; FV, *F. verticillioides*; FG, *F. graminearum.* Refer to **Table 1** for details about strain numbers. For hybrid maize, part of the data (control and single inoculations with FG1 and 2 and FV1 and 2) has already been described in Becker et al., 2014. The data is shown here for consistency with dwarf maize and for allowing the comparison to co-inoculations.

Volatile profiles presented a quite important quantitative variability within replicates of the same treatment (independent ears infected with the same fungus) whereas a qualitative variability in volatile composition was observed upon infection of different *Fusarium* species. This can be seen in the heatmaps of **Figure 5** that have been color coded to represent the concentration of infection biomarkers in dwarf and hybrid maize. As an example of qualitative variability, in both maize varieties, the volatile trichodiene was only detected from *F. graminearum* but never from *F. verticillioides.* In dwarf maize, hexan-1-ol was induced by FV3 (FV3 alone, FG3+FV3, FG4+FV3) compared to single inoculations with FG3 and FG4 (**Figure 5**).

The heatmaps of **Figure 5** further illustrate to which extent co-inoculations with two strains modulate emission of volatiles compared to single strains inoculations. For example in dwarf maize, inoculation with *F. verticilliodes* FV3 did hardly not induce sesquiterpenoids (i.e., (+)-longifolene, β-farnesene, α-muurolene, β-macrocarpene) whereas inoculation with *F. graminearum* FG4 did to a large extent. Simultaneous inoculation of dwarf maize ears with the two latter strains (FG4+FV3) lead to an intermediate situation where sesquiterpenoids were strongly induced in one ear/replicate only (similarly to FG4) but they were hardly not induced in the remaining four ears/replicates (similarly to FV3).

Overall the data highlight that hybrid and dwarf maize share common volatile markers mostly composed of sesquiterpenoids while they might differ in terms of volatiles belonging to other chemical classes.

## Discussion

Previously we demonstrated that maize ears infected with single *Fusarium* strains (i.e., *F. graminearum, F. subglutinans, F. verticillioides*) emitted specific volatiles, or disease biomarkers, which revealed the presence of the fungus even at a very early infection stages (Becker et al., 2014). If some volatiles (i.e., β-macrocarpene) seemed induced by all *Fusarium* species, others were species or strain specific (i.e., octan-3-ol was only induced by *F. verticillioides* and β-farnesene by *F. verticillioides* and *F. subglutinans*). Practically, these volatile biomarkers could potentially serve to identify infected ears and even to specifically identify the infecting *Fusarium* species. However, because in the field infection generally occurs with more than one *Fusarium* strain/species, it is essential to understand how competitive interactions influence the volatile blend of infected maize ears. Specifically our aim here was to understand how interaction between the two widely occurring species *F. graminearum* and *F. verticillioides* modulated volatiles emitted by maize ears.

Our data demonstrate that the volatile profile of maize ears infected with two *Fusarium* strains was dependant on the most competitive strain (**Figure 1**). Interspecific fungal interactions are driven by either interference- or exploitation-type competition (Peay et al., 2008). Interference competition in fungi involves direct interactions such as overgrowth or chemical competition whereas exploitation competition involves indirect negative effects resulting for example from the use of a common resource (Wicklow, 1981; Chatterjee et al., 2016). What strategy *Fusarium* strains use to compete among each other is unclear, and it might well be a mixture of chemical and exploitation competition. Mycotoxins such as tricothecenes and fumonisins have for long been candidates for chemical competition, however, we have recently demonstrated that they were not involved in such competition on maize ears (Sherif et al., unpublished).

Our data further demonstrate that volatile profiles of maize ears reflect disease severity regardless of the presence of one or more fungal pathogens. This conclusion was reached based on cluster analysis of the volatile profiles of infected maize ears (**Figure 2**), however, more powerful statistical models might be able to distinguish among ears infected with one or two pathogens. For example Thorn et al. (2011) analyzed the volatile profiles of 11 bacterial strains belonging to six species and, using a combination of similarity matrices, cluster analysis, and multidimensional scaling could successfully distinguish among strains belonging to the same species. From a practical perspective, however, cluster analysis on the maize volatile profile presented here provides useful information that can be used as a proxy to estimate disease severity and hence to potentially treat or sort infected kernels.

Detecting volatiles in real time under field conditions nevertheless remains a challenge essentially due to sensitivity issues. Sesquiterpenes are indeed released by plants in the range of 10–1000 of ng g^-1^_DW_ h^-1^ (Duhl et al., 2008) which is far below the detection limit of most portable instruments, and this emission highly fluctuates as a function of the plant’s circadian clock, but also further biotic and abiotic factors (Duhl et al., 2008; Loreto and Schnitzler, 2010). The latest generation of proton transfer-mass spectrometers (PTR-MS) might be sensitive enough for real time detection of these volatile biomarkers, even though their cumbersome size and high price remain a hindrance for the agro-business sector. One cheaper alternative might be provided by laser based photoacoustic systems as described in a recent review (Harren and Cristescu, 2013).

Overall using volatile sensing in the field to detect and possibly treat infected maize ears will require highly sensitive and affordable detection methods that operate reliably under variable weather conditions.

Our data also indicates that hybrid and dwarf maize share common volatile markers mostly composed of sesquiterpenoids while they differ in terms of other chemical classes of volatiles. These differences should be interpreted cautiously since we did not use the same fungal strains to infect hybrid and dwarf maize. Part of the differences observed among the two maize varieties might be attributed to the ability of either different *Fusarium* species/strains or of different maize cultivars to emit different volatiles. Variability in volatile profiles was indeed demonstrated for maize cultivars (Oluwafemi et al., 2012) and also for *Fusarium* species (Eifler et al., 2011). Nevertheless, the fact that a core volatile profile of sesquiterpenoids (β-macrocarpene, (+)-longifolene, β-farnesene, and trichodiene) was detected from both maize varieties is consistent with their ecological function. Indeed these volatiles can serve as building blocks for zealexins, metabolites involved in plant defense against fungal pathogens and insect pests (Huffaker et al., 2011). Interestingly in dwarf maize β-macrocarpene was not induced to detectable levels by single inoculations with *F. graminearum* FG3 and *F. verticillioides* FV3 nor by co-inoculation with the same strains (**Figure 5**). This suggests that similarly to what has been observed in maize root and stems and leaves (Köllner et al., 2008; Huffaker et al., 2011), β-macrocarpene might have been fully transformed into non-volatile zealexins.

A marked difference among *F. graminearum* strains was also detected for trichodiene, the volatile precursor of trichothecene toxins such as nivalenol and deoxynivalenol (Desjardins, 2006). In our study trichodiene was detectable from *F. graminearum* strains FG1, FG2, and FG4 but not from FG3 (**Figure 5**). These differences are supported by earlier quantifications of trichothecenes by the same strains. Indeed infection with FG3 results in the lowest trichothecene accumulation compared to the other strains [FG1 and FG2: >50 mg/kg; FG3 < 3.0 mg/kg; FG4: >400 mg/kg, Becker et al. (2014) and Sherif et al. (unpublished)]. This observation suggests that not only the presence/absence of specific infection markers but also their concentrations should be taken into account to estimate the infection level of maize ears.

Maize has developed an array of defense metabolites (phytoalexins) in response to fungal infections and attacks by herbivores. Zealexins, acidic sesquiterpenoid phytoalexins, accumulate to very high levels at infection sites of fungi and stem herbivores (Huffaker et al., 2011). Oxylipins, which result from the peroxidation of fatty acids by lipoxygenases (LOXs), are similarly involved in defense against pests and pathogens (Christensen et al., 2015) and the oxylipin 9-HOD (**Figure 1**) has been suggested as a biomarker for aflatoxin-resistance in maize lines (Wilson et al., 2001). The peroxidation of α-linolenic acid by 13-LOX yields 12-oxo-phytodienoic acid (12-OPDA) and downstream jasmonates, which includes the plant defense hormone jasmonic acid. By contrast, the peroxidation of α-linolenic and linoleic acid by 9-LOX lead to 10-oxo-11-phytodienoic acid (10-OPDA) and 10-oxo-11-phytoenoic acid (10-OPEA), which are involved in direct plant defense. Indeed, unlike jasmonates, 10-OPDA and 10-OPEA directly act as phytoalexins and display a significant phytotoxicity which highlights their involvement in localized cell death (Christensen et al., 2015).

In line with the latter studies, we observed earlier that several oxylipins and zealexins were induced upon infection of maize ears with single *Fusarium* strains and that disease severity correlated to oxylipins induction levels (Becker et al., 2014). The data presented with mixed inoculations in the current paper similarly indicates that disease severity correlates with the oxylipin 9-HOD in single and mixed inoculations (**Figure 1**). Linear correlation was, however, almost three times higher in single inoculations (*R*^2^ = 0.67, *p* < 0.05) compared to mixed inoculations (*R*^2^ = 0.23) suggesting that fungal competition might somehow compromise plant response. Interestingly compromised plant response in terms of repressed transcriptional factors (9- and 13-LOX) and reduced concentrations of zealexins were documented in maize ears and stalks infected by *F. verticillioides* under elevated CO_2_ concentration. Overall increased CO_2_ lead to increased susceptibility and repressed levels of zealexins (Vaughan et al., 2014). This highlights that more than one biotic or abiotic factor (CO_2_, competition) might compromise plant defense and begs for further studies to disentangle this complex interactions network.

Overall the data presented in this manuscript suggest that volatile profiles might be a good proxy for disease severity regardless of the fungal competition taking place in maize ears. With the appropriate sensitivity and reliability, volatile sensing thus appears as a promising tool for detecting fungal infection of maize ears under field conditions.

## Author Contributions

PK, MS, E-MB, and RS designed the work. MS, E-MB, and RS analyzed volatile profiles, disease severity and fungal biomass and interpreted the work with PK. IF and CH analyzed the oxylipins and interpreted the data together with E-MB, PK, RS, MS. MS and RS drafted the manuscript, which was critically revised by all coauthors.

## Conflict of Interest Statement

E-MB, RS, and PK declare having applied for a patent in 2013 describing the use of the volatile markers for identifying *Fusarium* infection in maize (patent application WO2013135889 A1). All the other authors declare that the research was conducted in the absence of any commercial or financial relationships that could be construed as a potential conflict of interest.
